# Noise Variation Characteristics of the Superconducting Gravimeter at Jiufeng Station in Wuhan (China)

**DOI:** 10.3390/s24237446

**Published:** 2024-11-22

**Authors:** Hang Li, Xiaodong Chen, Miaomiao Zhang, Xiaowei Niu, Jianqiao Xu, Heping Sun

**Affiliations:** 1 History, Culture and Tourism School, Fuyang Normal University, Fuyang 236037, China; 202409013@fynu.edu.cn; 2 State Key Laboratory of Geodesy and Earth’s Dynamics, Innovation Academy for Precision Measurement Science and Technology, Chinese Academy of Sciences, Wuhan 430077, China; zhangmm@apm.ac.cn (M.Z.); niuxiaowei@apm.ac.cn (X.N.); xujq@whigg.ac.cn (J.X.); heping@apm.ac.cn (H.S.); 3 University of Chinese Academy of Sciences, Beijing 100049, China

**Keywords:** Jiufeng station, SG, noise level, human activity, noise anomaly

## Abstract

The noise level of gravity stations is an important indicator for measuring the operating status of a station and is a prerequisite for evaluating whether the station’s observations can be used to extract weak geodynamic signals. With the continuous expansion of areas of human activity, gravity stations originally located in the wild may become increasingly closer to cities. Whether their noise levels change is an important issue that is worthy of attention. Based on power spectrum analyses and probability density function methods, the noise level of the superconducting gravimeter (SG) at Jiufeng station in Wuhan in the seismic frequency band of 0.001–0.04 Hz was calculated, and its time-varying characteristics were analyzed. The noise level of Jiufeng station did not change significantly before and after the lockdown of Wuhan due to the COVID-19 epidemic in 2020. No significant changes in the noise level were found before and after the official operation of Wuhan Metro Line 19 at the end of 2023. From October 2016 to April 2017, the noise level showed an abnormal trend of suddenly rapidly rising and then slowly declining, which was found to be caused by a tilt problem in the gravity sensor. Overall, in the seismic frequency band of 0.001–0.04 Hz, the noise level at Jiufeng station showed seasonal variation characteristics, and the noise was stronger in winter than in summer, which is consistent with the characteristics of Earth’s hum. Since January 2022, the noise level has shown an increasing trend year by year. The results of this study can provide an important reference for the operation of gravity stations and the extraction of weak geodynamic signals.

## 1. Introduction

The noise level is a key indicator for evaluating the quality of gravity station observations and the environment around them. It represents the ability of a gravimeter to detect weak geodynamic signals and can provide an important reference and basis for research on station site selection, instrument calibration, and the extraction of weak geodynamic signals [1,2,3,4]. The noise of an SG (superconducting gravimeter) mainly comprises two parts: the instrumental self-noise and the environmental noise around the station. The instrumental self-noise comes from the Brownian thermal motion generated by the damped mechanical oscillator in the gravity sensor, while the environmental noise depends on the geological structure and human activities around the station. According to a study by Rosat [5], in the seismic frequency band, the theoretical value of thermal noise generated by a damper is consistent with the observed values obtained using the three-channel correlation analysis method. The source of self-noise of SGs in the seismic frequency band is known, but there is a lack of analysis on the influence of the environmental changes around the station. Only when the environment changes greatly is it easy to find the influence on observations. Gravity stations are usually located in the wild, but with the development of modern cities, the area of human activity has greatly increased, and stations that were previously far away from human activities have become closer and closer to them. When a station is located inside a city, the human activities around it will have a great impact on the noise level of SGs [1]. When the COVID-19 epidemic broke out in early 2020, Wuhan implemented the strictest lockdown measures, and human activities in the city were greatly reduced, which provided us with a rare opportunity to conduct such research. There is an SG station named Jiufeng in Wuhan. By comparing the changes in the noise level of the SG at Jiufeng station before and after the city was locked down, we can quantitatively analyze the influence of human activities around Jiufeng station on gravimeter observations.

The lockdown measures triggered by the COVID-19 epidemic not only interrupted people’s lives and economic development but even affected the records of seismic stations around the world. The authors of an article published in *Science* pointed out that in the first half of 2020, during the period when social distancing and other related measures were taken to slow the spread of the COVID-19 epidemic in human society, the noise level recorded by global seismometers dropped significantly, with the largest drop being up to 50% (the noise levels in mainland China dropped most significantly) [6]. This phenomenon provided an unprecedented opportunity for the detection of weak geodynamic signals submerged in background noise, and the strong correlation between noise levels and human activities also indicates that seismic technology can become a method for studying population dynamics [7,8,9,10]. Hong et al. studied the relationship between daytime high-frequency noise recorded with seismometers and economic development [11]. Their study found that daytime noise of 5–15 Hz recorded with seismometers shows a high correlation with human activities, including transportation and factory operations related to economic activities.

In previous studies, the frequency of the noise level drop caused by the lockdown measures recorded with seismometers was concentrated above 1 Hz, but was the low-frequency noise less than 1 Hz also affected? In studies based on seismic observations, researchers have found that the noise generated by human activities is mainly concentrated at 1–40 Hz [11,12,13]. Occasional studies have also found that public transportation such as railways and subways clearly interfere with the 0.01–0.05 Hz frequency band in seismic records [14]. Sheen et al. found that some seismic stations in South Korea recorded noise excited by railways 2 km away in the frequency band of 0.01–0.05 Hz. Although the observed vibration energy excited by railways is relatively weak, it is still about 20 dB higher than the noise level on quiet days [14]. For low-frequency noise, do human activities also contribute? So far, studies based on seismic observations have not been conclusive.

SGs can be regarded as vertical seismometers and can be used to record low-frequency seismic noise [15,16,17,18]. Therefore, is the low-frequency noise recorded by SGs also related to human activities? In 1997, Banka analyzed the observations of the SG-T004 when it was placed in the lobby on the first floor of the experimental building of the Wuhan Institute of Geodesy and Geophysics. He found that the noise level in the seismic frequency band (including the normal-mode frequency band) observed by the SG was evidently disturbed by human activities, showing the characteristics of strong noise during the daytime and weak noise at nighttime [1]. Li et al. found a drop in the SNM (seismic noise magnitude) values before and after the Wuhan lockdown in 2020 based on the observations of the gPhone gravimeter installed inside the campus of the Chinese University of Geosciences [19]. The results showed that the noise level during the Wuhan lockdown in 2020 was lower than that during the same period in 2019. In both studies, the gravimeters were installed inside buildings with intensive human activities, so it is impossible to determine whether the above conclusions can be applied to stations that are away from human activities. The Wuhan lockdown provided a great opportunity to study whether there is a correlation between the ambient noise observed by the SG at Jiufeng station and human activities. The strict lockdown measures in Wuhan made Jiufeng station a quieter environment than other stations in the world, and it was easier to detect changes in the noise level of the station before and after the lockdown.

A quiet environment is crucial for high-precision gravity observations, such as those made with SGs. In recent years, some studies have attempted to place gravimeters in underground environments in order to seek ultra-quiet environments and lower noise levels. The difference between surface and underground environments is mainly due to the impact of human activities. Rosat et al. calculated the noise level of the iOSG-24 SG in a low-noise underground laboratory (LSBB) in France and found that the combination of the SG and the underground environmental conditions made the station one of the quietest SG stations in the world [20]. Based on the gravimeter observations of an underground laboratory in Huainan, China, Sun et al. calculated the noise levels in two different environments: surface and underground (−848 m) [21]. They found that the underground noise level was significantly lower than that on the ground, which shows that the underground ultra-quiet observational environment has advantages in detecting weak signals. Based on observations from 2019 to 2021 by two SGs installed at the surface and at an underground depth of 520 m in the LSSB in France, Rogister et al. found that the difference in depth of 520 m had little influence on the results of semidiurnal tide gravity observations [22]. The error of the calibration factors had a greater impact on the magnitude of the observed gravity tides than the difference in depth.

Jiufeng station is located in the suburbs of Wuhan and has a history of more than 20 years. With the rapid development of urban construction in recent years, the urban area of Wuhan has been expanding outward. Has the traffic near Jiufeng station, such as the recent operation of Metro Line 19, affected the observations of the SG at Jiufeng station? Does the low-frequency noise below 1 Hz partly come from human activities? Is the performance of the SG at Jiufeng station as stable as before? Relevant research on these issues is conducted in this study.

In this study, the noise level of the SG at Jiufeng station in Wuhan over the last decade is calculated. The long-term evolution characteristics of the noise level in the seismic frequency band of 0.001–0.04 Hz and the changing characteristics of the noise level before and after time nodes such as the Wuhan lockdown in 2020 and the official operation of Metro Line 19 in 2023 are studied.

## 2. Data and Methods

### 2.1. Data

Jiufeng station (National Geodetic Observatory) is located on the northern ridge of Shimenfeng in the suburbs of Wuhan. It is the only international gravity tide benchmark station on the Chinese mainland. It is also an important experimental and research base for comparing observations from different types of gravimeters and for verifying the solid Earth tide and ocean tide models on the Chinese mainland [23]. In 1997, the GWR (Goodkind–Warburton–Reineman) C032 (Number) SG was successfully installed in the gravity observation room at Jiufeng station, which was about 25 km away from the urban area of Wuhan at that time. The gravity observation room was built by digging into the north side of the mountain using an integral frame structure and then backfilling [24]. The observation pier of the SG is located on bedrock and is cast with cement. The SG station has a very quiet environment, and its high-quality observations are widely used in research on gravity tides, non-tidal changes, and geodynamic problems. In 2013, the OSG-065 (observatory superconducting gravimeter No. 065) SG was installed at Jiufeng station to replace the GWR C032, which had stopped operating, and the OSG-065 has been in operation ever since. In order to ensure that the observations used in our study are all from the OSG-065, 1 s sampled observations from January 2014 to May 2024 were selected. This time period covers important events such as the Wuhan lockdown in 2020 and the operation of Metro Line 19 in 2023, satisfying the requirements of the study. The data gaps are given in Table 1.

### 2.2. Methods

#### 2.2.1. Power Spectrum Analysis

In this study, the power spectrum analysis method was used to calculate the noise level of the SG at Jiufeng station in Wuhan. First, the solid Earth tide signals in the observations were deducted, and then a Fourier transform was performed according to the following formula:

(1) $$Y\left(f,T\right)=\int_{0}^{t}y\left(t\right)e^{-i2\pi ft}dt$$

where y(t) are gravity residuals, T is the length of the gravity residuals, and f is the frequency.

The Fourier component at the discretized frequency is defined as follows:

(2) $$Y_{k}=\frac{Y\left(f_{k},T\right)}{\Delta t}$$

In the formula, $f_{k}=k/N\Delta t$, k=1,2,…,N−1, Δt is the sampling interval, N is the number of sampling points, and N=T/Δt. The power spectrum formula defined by the Fourier components can be expressed as

(3) $$P_{k}=\frac{2\Delta t}{N}{\left|Y_{k}\right|}^{2}$$

In order to study the changes in noise over time, we calculated the root mean square (rms) of the time-domain gravity residuals based on the power spectrum. The Pasval formula was used in the calculation [6]. The final formula is shown as follows:

(4) $$d_{rms}\left(t\right)=\sqrt{\int_{f_{\min}}^{f_{\max}}P\left(f\right)df}$$

P(f) is the power spectral density at the frequency f. $f_{\max}$ and $f_{\min}$ are the range of the frequency band. The discretized form of Equation (4) is used in the calculation. In this study, $f_{\max}=0.04\;\mathrm{Hz}$ and $f_{\min}=0.001\;\mathrm{Hz}$.

#### 2.2.2. Probability Density Function

In order to perform statistical analyses for the results of the power spectrum, we used the probability density function method [25]. This method resamples the above power spectrum estimation results at 1/8 octave frequency intervals over the frequency range. This processing step greatly reduces the number of frequency points and significantly reduces the amount of calculation. In this way, the average power spectrum value between $T_{s}$ and $T_{s}=2T_{l}$ is calculated (the power spectrum unit is converted to dB) as the power spectrum value at the center period $T_{c}=\sqrt{T_{s}T_{l}}$. Then, $T_{c}$ can be evenly distributed on the logarithmic axis. Then, $T_{s}$ is increased with a 1/8 octave step size ($T_{s}=T_{s}*2^{0.125}$), and the average power spectrum in the next period interval is calculated. In the new period interval, $T_{l}$ and $T_{c}$ are also recalculated. The power spectrum values distributed in the interval of $T_{s}$ and $T_{l}$ are averaged, and then the calculation process is continued until the longest period.

In the calculations, a sliding window is used to calculate the power spectrum density of gravity residuals, where the window width is 1 h and the sliding step is 30 min. In this way, all of the data are divided into a large number of segments, and then the power spectrum results of these 1-h segments are statistically analyzed. The probability density function value at a given central period can be estimated as follows:

(5) $$P\left(T_{c}\right)=N_{PT_{c}}/N_{T_{c}}$$

In Formula (5), $N_{PT_{c}}$ represents the number of power spectrum values falling within the 1 dB width interval of the vertical axis at the center period $T_{c}$ and the vertical axis ranges from −200 dB to −80 dB. $N_{T_{c}}$ represents the number of power spectrum values along the vertical axis at the center period $T_{c}$.

#### 2.2.3. Continuous Wavelet Transform

In order to ensure the reliability of the calculation results, we also adopted the continuous wavelet transform method. The calculation formula is as follows:

(6) $$W\left(a,b\right)=\frac{1}{\sqrt{a}}\int_{-\infty }^{\infty }y\left(t\right)\psi^{*}\left(\frac{t-b}{a}\right)dt$$

where y(t) are gravity residuals, and a and b are the wavelet scale and the translation parameter, respectively. W(a,b) is the wavelet coefficient. $\psi^{*}$ is the complex conjugate of the Morlet wavelet.

## 3. Results

### 3.1. The Noise Levels Before and After the Lockdown of Wuhan in 2020

**Figure 1:**
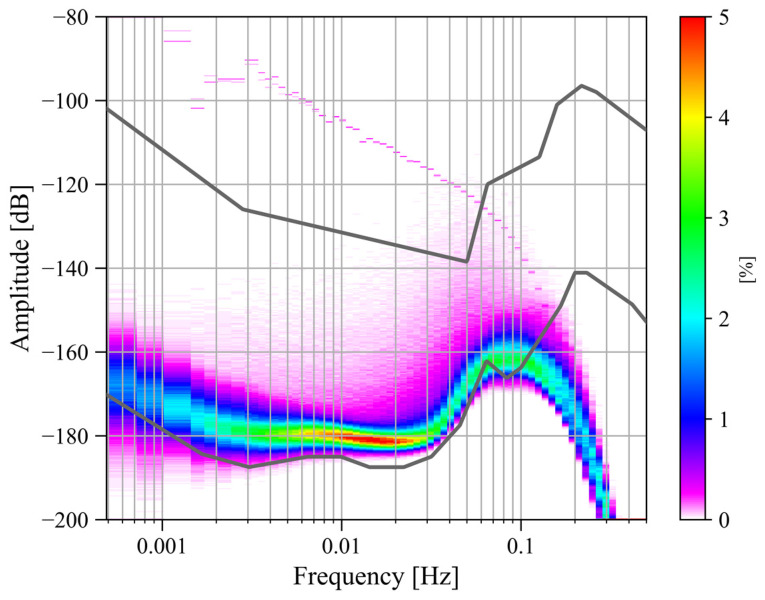
Noise probability density distribution from January 2019 to December 2020 (the upper and lower gray curves represent the new high-noise model and the new low-noise model, respectively).

Due to the outbreak of the COVID-19 pandemic, Wuhan was locked down for 76 days from 23 January to 8 April 2020, during which most human transportation and construction activities were prohibited. In order to analyze whether human activities would affect the noise level of the SG at Jiufeng station, we processed the observations before and after the lockdown. First, based on the power spectrum analysis and probability density function methods described above, we calculated the noise level using gravity residuals from January 2019 to December 2020. We focused on the noise in the seismic frequency band of 0.001–0.04 Hz. The final noise probability density distribution is shown in Figure 1.

The two gray curves in Figure 1 represent the new low-noise model (NLNM) and the new high-noise model (NHNM) [26]. It can be seen in Figure 1 that the maximum noise power is located around 0.1 Hz. The noise near this frequency corresponds to microseisms in seismology. According to the results based on seismic observations, microseisms can be divided into the primary microseisms of 0.04–0.1 Hz and the secondary microseisms of 0.1–0.2 Hz. Therefore, in Figure 1, there should be two peaks corresponding to these two types of microseisms. The primary microseisms are caused by the coupling of ocean swells and the inclined seafloor at shallow coasts [27]. The secondary microseisms are mainly excited by the coupling of the second-order pressure on the seafloor and the solid Earth. This second-order pressure, which does not attenuate with depth, is caused by the standing waves formed by the superposition of ocean waves with the same frequency and opposite propagation directions in the deep ocean [28,29,30,31,32]. In Figure 1, only the peak of the primary microseisms is observed. This is because the data acquisition system of the SG performs low-pass filtering when converting analog signals into digital signals to prevent spectrum aliasing, which causes the noise energy in the frequency band of microseisms to be filtered out, so the peak of the secondary microseisms cannot be seen in Figure 1. Another feature in Figure 1 is that the noise in the frequency band of 0.001–0.04 Hz is mainly concentrated around −180 dB. Except for the secondary microseisms affected by low-pass filtering, the noise energy in 0.001–0.04 Hz is the smallest in the entire frequency range, and the noise distribution is relatively uniform. The noise in this frequency band is called Earth’s hum, and it appears as surface waves and free oscillations. According to studies by seismologists and oceanographers, the excitation mechanism of Earth’s hum is similar to primary microseisms, that is, the coupling excitation of ocean waves and seafloor topography. The difference is that Earth’s hum is excited by the coupling of ocean infragravity waves and the seafloor topography in the deep ocean [27,33,34]. In addition, the coupling between atmospheric disturbances in the vicinity of volcanic sources caused by volcanic eruptions and the solid Earth is another important source of excitation [35,36,37]. Since the noise level in the frequency band of 0.001–0.04 Hz is flat and is not affected by the analog low-pass filter in the data acquisition system, the frequency band concerned in this study is defined as 0.001–0.04 Hz.

**Figure 2:**
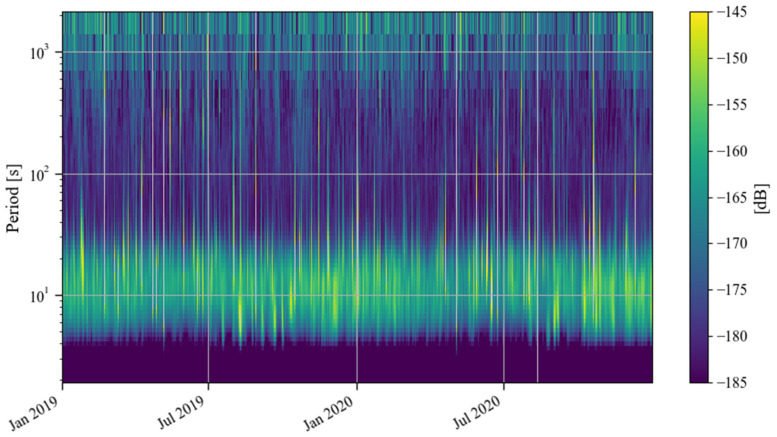
Time–frequency spectrum of noise from January 2019 to December 2020 (the blank areas indicate noise values larger than −145 dB).

**Figure 3:**
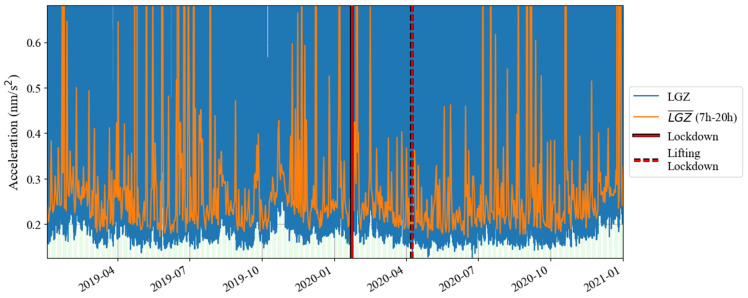
Daily noise variations from January 2019 to December 2020 (the blue line denotes hourly noise, and the orange line denotes the median noise during the daytime).

**Figure 4:**
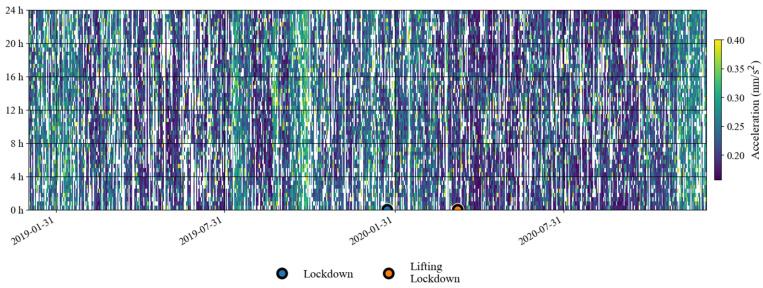
Hourly noise variation from January 2019 to December 2020 (the blank areas indicate noise values larger than 0.4 nm/s^2^).

**Figure 5:**
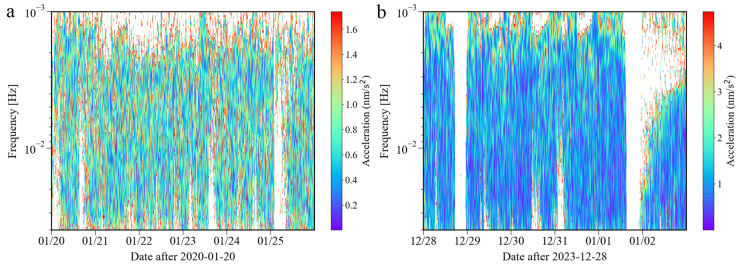
Wavelet transform spectrum of the noise during (**a**) the lockdown measures and (**b**) the opening of Metro Line 19 (the blank areas indicate noise values larger than (**a**) 1.7 nm/s^2^ and (**b**) 4.6 nm/s^2^).

Figure 2 shows the time–frequency spectrum of the noise of the SG at Jiufeng station from January 2019 to December 2020. The blank area in this figure indicates that the noise power is greater than −145 dB. As can be seen in Figure 2, there is a high level of power in the 5–20 s period range, which corresponds to microseisms. The Earth’s hum in the frequency band of 0.001–0.04 Hz, which is of interest in this study, is shown as a continuous low power in Figure 2. Due to the interference of high noise in the frequency band of microseisms, no features can be seen for Earth’s hum in this figure. Before and after the lockdown of Wuhan in 2020, no reduction in noise was observed. Note that the UTC time zone is used in Figure 1 and Figure 2. To calculate the local daytime (defined as 7 a.m. to 8 p.m. in local time) noise change in Wuhan, the UTC time is converted into the local time in Figure 3, Figure 4 and Figure 5. The same type of figure is treated in this way in the following.

According to the results in Figure 1 and Figure 2, based on Formula (4), we calculated the change in noise (root mean square of gravity residual) over time in the frequency band of 0.001–0.04 Hz from January 2019 to December 2020; the result is shown in Figure 3. In Figure 3, the blue curve represents the result (1 h corresponds to one noise value), while the orange curve represents the median power spectrum of the noise in each daytime period (7 a.m. to 8 p.m. in local time). The channel of the gravity observations is denoted by ‘LGZ’. The weekdays are also marked by the light green shadows in Figure 3. It can be seen that the changing trends represented by the lower envelope of the orange curve and the blue curve are consistent, so we regard the orange curve as the main result. The red vertical solid lines and dotted lines in Figure 3 indicate the times when Wuhan went under lockdown and when the lockdown was lifted in 2020, respectively. It can be seen that the noise in 0.001–0.04 Hz did not decrease significantly before and after the lockdown. According to the trend of the lower envelope of the orange line, it can be seen that in 0.001–0.04 Hz, the SG at Jiufeng station had strong noise in winter and weak noise in summer, which is consistent with the characteristics of the magnitude of the Earth’s hum as revealed by seismic observations [38]. The excitation source of Earth’s hum is located in the North Pacific in winter and in the Antarctic Sea in summer [39]. Since Jiufeng station is located in the northern hemisphere, it is closer to the North Pacific, so the noise appears to be stronger in winter than in summer.

The above analysis shows that the lockdown measures that occurred in January 2020 did not have a significant impact on the noise level of the SG at Jiufeng station in the seismic frequency band of 0.001–0.04 Hz. Figure 3 shows the seasonal variation characteristics, revealing that in addition to the instrument self-noise (white noise) caused by Brownian motion in the damper, the remaining noise in this frequency band was mainly from Earth’s hum. Since no noise reduction was found during the Wuhan lockdown period, as shown in Figure 3, this indicates that the SG observations at the Jiufeng station far away from the urban area are likely not affected by human activities. In order to further rule out the possibility that the noise in this frequency band is related to human activities, the hourly changes in noise during the lockdown of Wuhan were plotted, with one noise value obtained every half hour. The final result is shown in Figure 4. The blue and orange dots denote the times of the lockdown and the lifting of the lockdown, respectively. From Figure 4, we also identify that the noise was strong in winter and weak in summer. It can be seen that the noise in the frequency band of 0.001–0.04 Hz continued to exist within 24 h of one day, and there was no clear difference in the strength of the noise during the daytime and nighttime. Since human activities are mainly concentrated during the daytime, the above results further indicate that the noise in this frequency band was likely not affected by human activities. The results of this study are different from those of Li et al. [19]. This is mainly because the gPhone gravimeter that they used was located inside a human activity area, so it was more susceptible to interference from human activities. This is similar to the result from Banka [1]. In our study, Jiufeng station is far away from urban areas and is not easily affected by human activities, which leads to different conclusions.

The spectrum results of the noise calculated based on wavelet transform are given in Figure 5a. Because the sample interval of the observations is 1 s, considering the amount of calculation, only the results of a few days before and after the time (23 January 2020) of the lockdown measures in Wuhan were calculated. From Figure 5a, the phenomena of noise reduction cannot be found. The result of the wavelet transform further confirmed that the noise level of the SG at Jiufeng station was not affected by the lockdown measures in Wuhan.

### 3.2. The Noise Level Before and After the Operation of Wuhan Metro Line 19

**Figure 6:**
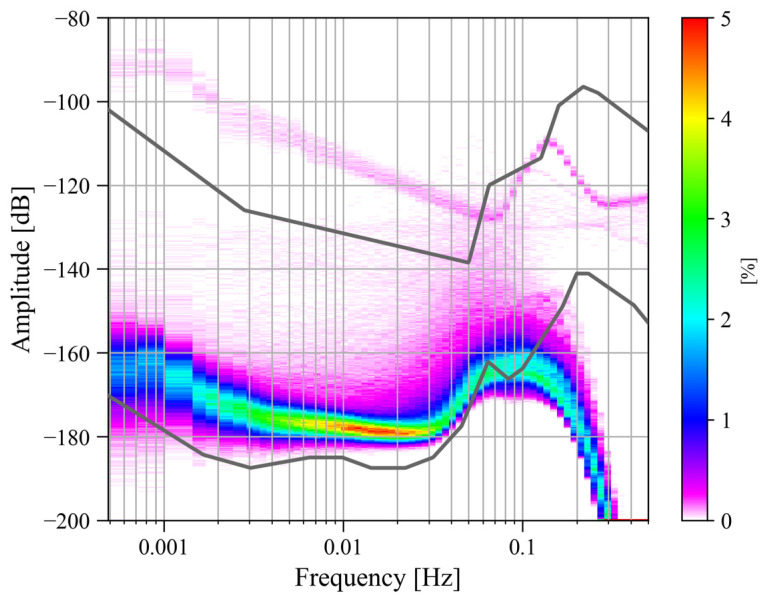
Noise probability density distribution from May 2023 to April 2024 (the upper and lower gray curves represent the new high-noise model and the new low-noise model, respectively).

If the reason why the noise level of the SG at Jiufeng station did not change before and after the Wuhan lockdown in January 2020 is that the station is far away from human activity areas, then would the recently operational Wuhan Metro Line 19 affect the noise level in the seismic frequency band of 0.001–0.04 Hz? Wuhan Metro Line 19 was officially operational on 30 December 2023, and the shortest distance between the subway line and Jiufeng station is only a few hundred meters. When the train runs close to the station, will it affect the observations of the SG? In order to answer this question, the noise variations before and after the operation of Line 19 are analyzed in this study. We first calculated the noise probability density distribution from May 2023 to April 2024, and the results are shown in Figure 6.

**Figure 7:**
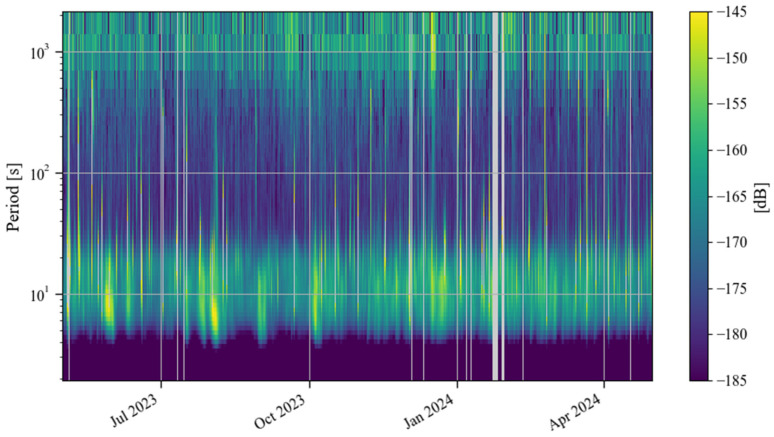
Time–frequency spectrum of noise from May 2023 to April 2024 (the blank areas indicate noise values larger than −145 dB or data gaps in Table 1).

It can be seen in Figure 6 that from May 2023 to April 2024, the noise level in 0.001–0.04 Hz was greater than −180 dB, which was higher than the noise level before and after the lockdown in Wuhan, as shown in Figure 1. Moreover, in the frequency band of 0.001–0.04 Hz, as the frequency increased, the noise tended to gradually decrease. In addition, in Figure 6, there are a small number of data segments with abnormally high noise power (between −130 dB and −90 dB), which came from observational anomalies of the instrument. This part of the data anomaly corresponds to a data anomaly at the end of January 2024 in the time–frequency spectrum shown in Figure 7 (this noise anomaly is shown as a blank in Figure 7).

**Figure 8:**
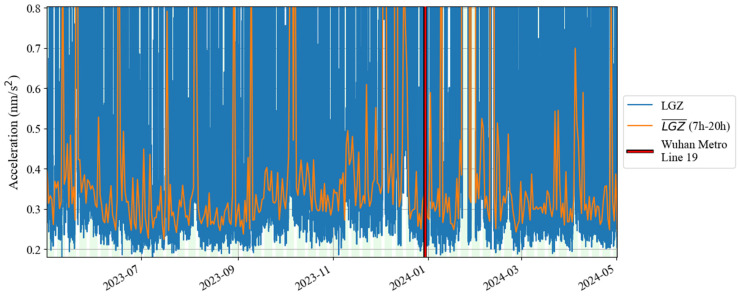
Daily noise variations from May 2023 to April 2024 (the blue line denotes the hourly noise, and the orange line denotes the median noise during the daytime).

**Figure 9:**
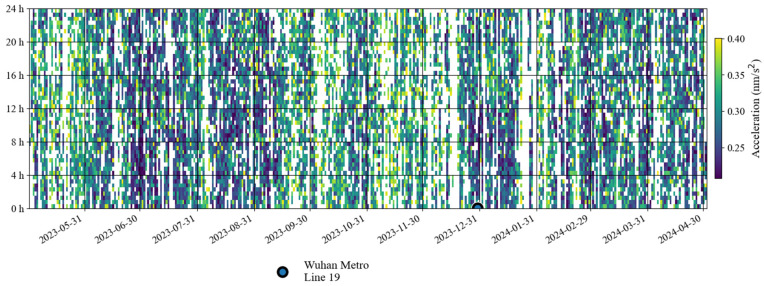
Hourly noise variations from May 2023 to April 2024 (the blank areas indicate noise values larger than 0.4 nm/s^2^ or data gaps in Table 1).

Figure 7 shows the noise time–frequency spectrum from May 2023 to April 2024. This image shows noise characteristics similar to those in Figure 2. It can be seen in the figure that the power of the microseisms was the strongest. Sometimes, due to the influence of surface waves excited by earthquakes, the power is significantly enhanced. In order to further analyze the changing trend of noise over time in the frequency band of 0.001–0.04 Hz that this study focuses on, we also calculated the daily and hourly changes in the root mean square of the gravity residuals based on Formula (4). Figure 8 shows the daily changes in noise from May 2023 to April 2024. According to the changing trend of the median noise during the daytime (7–20 h) represented by the orange curve, it can be seen that the noise level in winter was slightly higher than that in summer. The red vertical line in Figure 8 indicates the operation time of Wuhan Metro Line 19. As shown in Figure 8, no noise enhancement was found after the subway began operation. Figure 9 shows the hourly noise change results. The blue dot marks the operation time of Wuhan Metro Line 19. Similarly, as shown in Figure 9, no noise enhancement phenomenon was found after the subway began operation. It can also be seen that for the noise in 0.001–0.04 Hz, there was no apparent difference in noise between daytime and nighttime. The above results indicate that the operation of Wuhan Metro Line 19 did not have a significant impact on the noise level of the SG at Jiufeng station. This conclusion can also be obtained from the results of the wavelet transform shown in Figure 5b.

## 4. Discussion

### 4.1. Variations in the Noise Level in the Last Decade

As mentioned above, neither the lockdown measures nor the operation of Metro Line 19 had a significant impact on the noise level in the seismic frequency band of 0.001–0.04 Hz. By referring to the economic data, we find that the GDP (gross domestic product) of Wuhan in 2023 was twice that in 2014, which indicates that the increase in urban construction has not stopped. Thus, looking at a longer time scale, that is, with the development of the urban construction of Wuhan in the last ten years, has the noise level of the SG at Jiufeng station changed? In order to obtain the long-term trend of noise levels, observations from January 2014 to May 2024 were selected. The probability density distributions of the noise in the seismic frequency band were calculated, and the noise evolution characteristics over time were analyzed.

**Figure 10:**
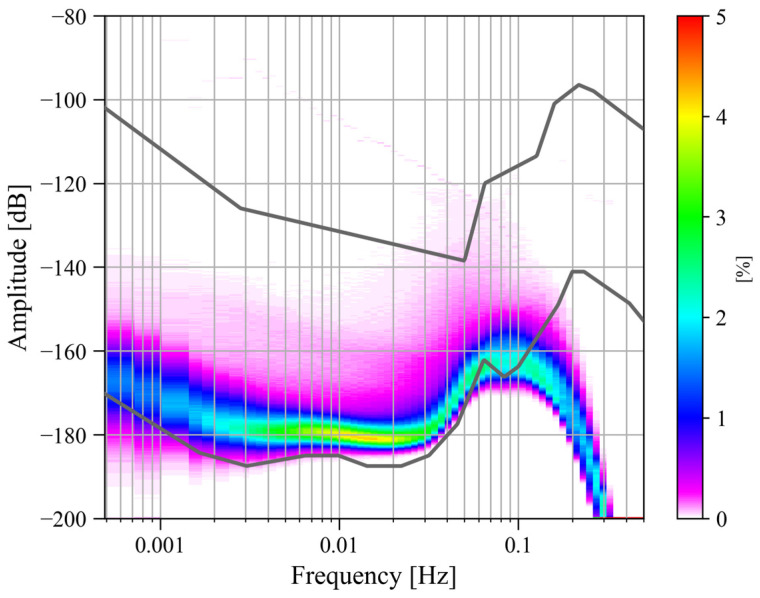
Noise probability density distribution from January 2014 to May 2024 (the upper and lower gray curves represent the new high-noise model and the new low-noise model, respectively).

Figure 10 is the noise probability density distribution from January 2014 to May 2024. Overall, in the last ten years, the noise in 0.001–0.04 Hz was mainly concentrated around −180 dB and did not change with frequency. This is similar to the results from January 2019 to December 2020 in Figure 1, but it is different from the results from May 2023 to April 2024 in Figure 6. Comparing the above results, it can be found that the noise in 0.001–0.04 Hz in the last decade was about −180 dB most of the time, but the noise level has recently increased (results for 2023–2024 in Figure 6).

**Figure 11:**
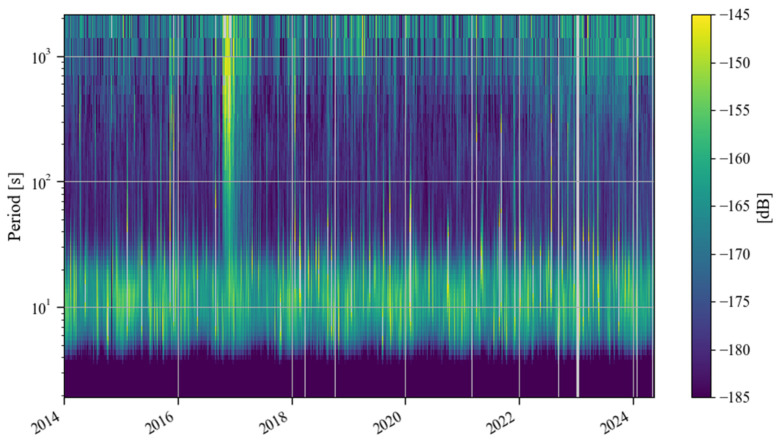
Time–frequency spectrum of noise from January 2014 to May 2024 (the blank areas indicate noise values larger than −145 dB or data gaps in Table 1).

**Figure 12:**
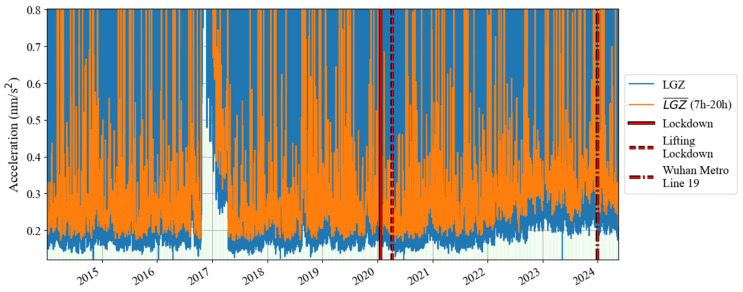
Daily noise variations from January 2014 to May 2024 (the blue line denotes hourly noise, and the orange line denotes the median noise during the daytime).

Figure 11 shows a time–frequency spectrum of the noise in the last decade. It can be seen that from the second half of 2016 to the first half of 2017, there was a strong noise anomaly in 0.001–0.04 Hz. This feature is easier to find in Figure 12. In addition to these noise anomalies, it can be seen in Figure 12 that since January 2022, the noise of the SG at Jiufeng station has become significantly higher. It can also be seen that the noise in 0.001–0.04 Hz shows seasonal variation characteristics, that is, the noise is strong in winter and weak in summer. These characteristics of seasonal variation have been pointed out in previous results. It can be seen in Figure 12 that this feature still existed even at the stage when the noise suddenly became stronger after January 2022.

**Figure 13:**
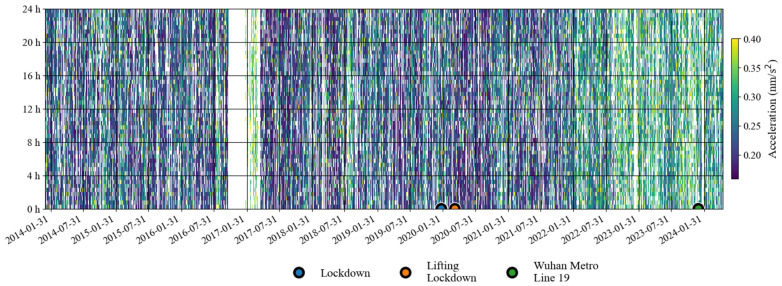
Hourly noise variations from January 2014 to May 2024 (blank areas indicate noise values larger than 0.4 nm/s^2^ or data gaps in Table 1).

In order to avoid the strong noise anomaly appearing in 2016–2017 affecting the display of noise features, we set the results corresponding to the noise anomaly time period as blank areas; the subsequent hourly noise variation results are given in Figure 13. In this figure, the most striking feature is that the noise suddenly became stronger in January 2022, and there has been a strong noise state since then. This can also be seen in Figure 12. The difference is that Figure 13 shows that the strong noise does not change between daytime and nighttime, which means that the noise has nothing to do with human activities that only occur during the daytime. It can also be seen that there has been a weak seasonal noise variation in the last ten years. This phenomenon is consistent with the seasonal variation characteristics of Earth’s hum.

### 4.2. Analysis of Noise Anomalies Between 2016 and 2017

**Figure 14:**
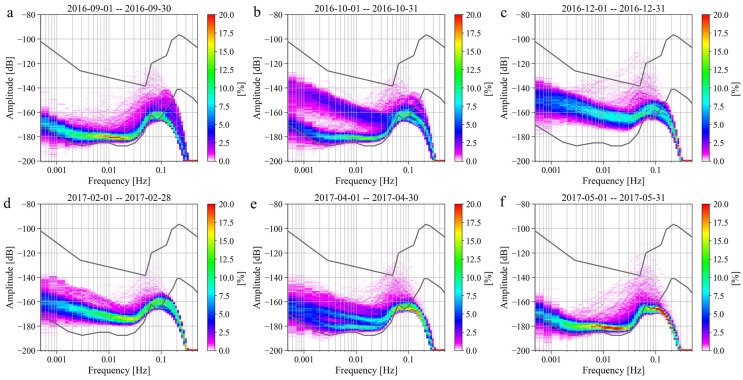
Monthly variations in the noise probability density distribution in (**a**) September 2016, (**b**) October 2016, (**c**) December 2016, (**d**) February 2017, (**e**) April 2017 and (**f**) May 2017 (the upper and lower gray curves in the figure represent the new high-noise model and the new low-noise model, respectively).

**Figure 15:**
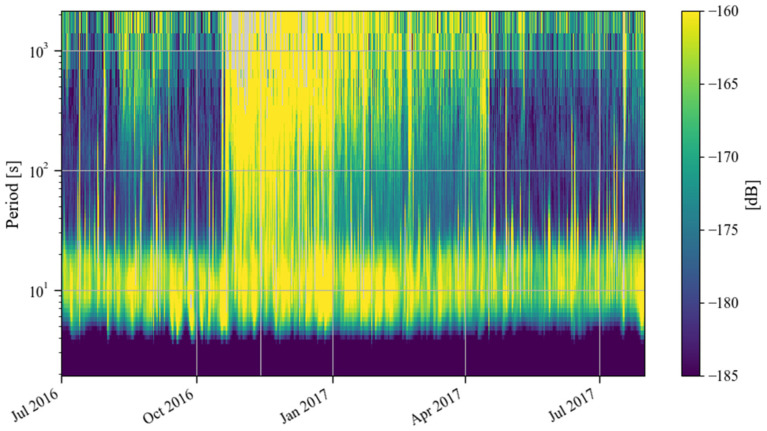
Time–frequency spectrum of noise from July 2016 to July 2017 (the blank areas indicate noise values larger than −160 dB).

In the previous section, we found that there was a strong noise anomaly from the second half of 2016 to the first half of 2017. In this section, we analyze this issue in detail. First, we calculate the monthly probability density distribution of the noise of the SG at Jiufeng station from July 2016 to July 2017. The results are shown in Figure 14. As can be seen in this figure, the noise level in 0.001–0.04 Hz was normal in September 2016 and was mainly concentrated around −180 dB. However, starting in late October 2016, the noise suddenly increased and remained high until late April 2017, when the noise level gradually returned to normal. From the time–frequency spectrum shown in Figure 15, it can be seen that the intensity of the noise anomaly was close to or even stronger than microseisms.

**Figure 16:**
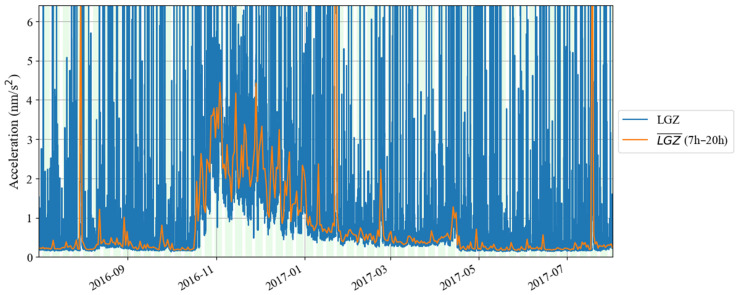
Daily noise variations from July 2016 to July 2017 (the blue line denotes the hourly noise, and the orange line denotes the median noise during the daytime).

**Figure 17:**
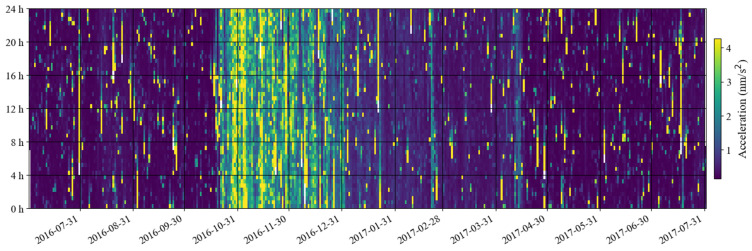
Hourly noise variations from July 2016 to July 2017 (the blank areas indicate noise values larger than 4.27 nm/s^2^).

**Figure 18:**
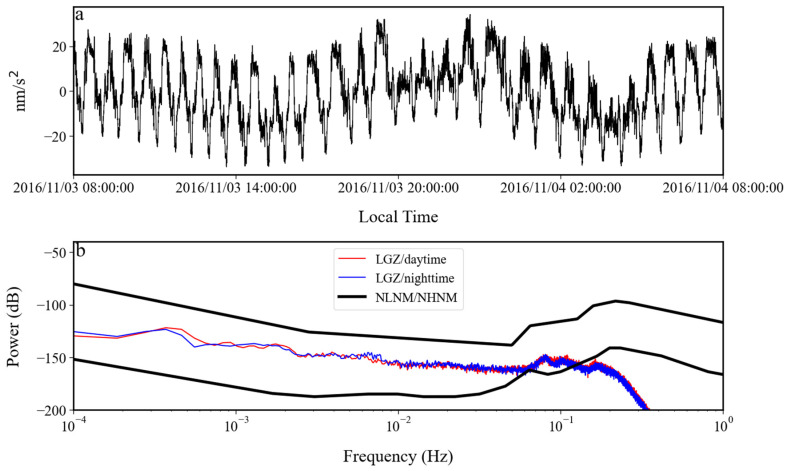
(**a**) Gravity residues and (**b**) the corresponding spectrum on 3 November 2016.

In order to understand the variation process of the above noise anomaly, based on Formula (4), we calculated the root mean square of hourly gravity residuals from July 2016 to July 2017. The results are shown in Figure 16 and Figure 17. It can be seen in these two figures that in October 2016, the noise had a rapid increase. The noise intensity gradually decreased from November 2016. In January 2017, the speed of noise reduction gradually slowed down. It was not until the end of April 2017 that the noise intensity returned to normal levels. In Figure 17, no variation in the intensity of the noise anomaly with daytime and nighttime is found, which indicates that the noise anomaly may have come from the instrument itself. In order to further analyze this issue, the raw gravity time series were checked. It was found that the noise anomaly began to appear on 24 October 2016 and lasted until 17 April 2017. Figure 18 shows the gravity residues and corresponding spectrum on 4 November 2016. A peak with a frequency of 0.4 mHz can be seen in the spectrogram, which corresponds to this noise anomaly. As can be seen in Figure 18, when the frequency was greater than 0.4 mHz, the noise power gradually decreased with the increase in frequency.

**Figure 19:**
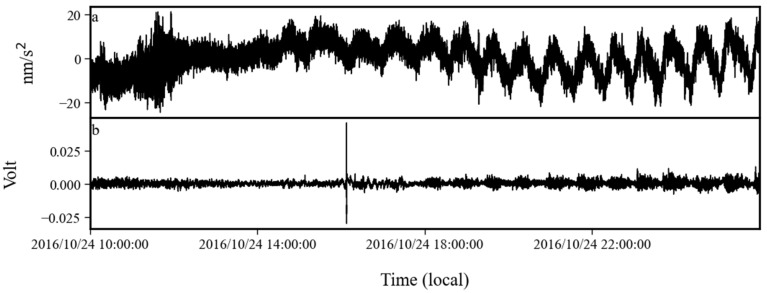
(**a**) Gravity and (**b**) tilt observations on 24 October 2016.

**Figure 20:**
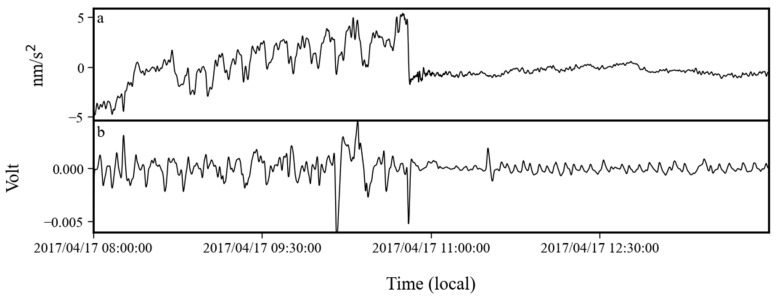
(**a**) Gravity and (**b**) tilt observations on 17 April 2017.

By analyzing the auxiliary data from this time period, we found that the noise anomaly was caused by a tilt problem in the sensor. In Figure 19 and Figure 20, we plot the time series of gravity and tilt (voltage measured by the tilt sensor) on 24 October 2016 and 17 April 2017. Figure 19 shows the raw data, and Figure 20 gives the data after low-pass filtering with a cutoff frequency of 0.01 Hz to eliminate the interference of earthquakes. From these two figures, it can be seen that the period of the gravity noise anomaly was about 0.4 mHz, and the start and end times of the anomaly gravity signal were consistent with the anomaly signal in the tilt observation. The tilt caused the axis of the sensor to move away from the direction of the gravitational acceleration. At this time, there was a non-zero angle between the direction of gravitational acceleration and the axis of the sensor, and there was a component of gravity acceleration along the axis. This angle changed with time, which also caused the observations of the gravity sensor to change with time, i.e., noise anomaly. Its period was consistent with the period of the tilt. The above evidence demonstrates that the noise anomaly from October 2016 to April 2017 was caused by the tilt in the gravity sensor.

## 5. Conclusions

The noise variation characteristics of the OSG-065 at Jiufeng station in the seismic frequency band of 0.001–0.04 Hz are analyzed in this study. By analyzing the data during the lockdown in Wuhan in 2020, we found that the noise level (0.001–0.04 Hz) did not change significantly. In addition, after the beginning of the operation of Wuhan Metro Line 19 at the end of 2023, no increase in the noise level was observed. A strong noise anomaly, occurring from October 2016 to April 2017, was found in our investigation. The main feature of this was that the noise level suddenly increased from October 2016 and then gradually dropped to normal levels in April 2017. This noise anomaly did not exhibit different characteristics in daytime and nighttime. With the help of auxiliary data, it was found that the noise anomaly was caused by the tilt of the gravity sensor. Through the analysis of observations in the last ten years, it was found that since January 2022, the noise level of the SG at Jiufeng station has always been high. The noise in 0.001–0.04 Hz shows seasonal variation characteristics; that is, the noise is strong in winter and weak in summer. This result indicates that the noise is mainly from Earth’s hum, which is generated by the coupling of infragravity waves in the deep ocean and the seabed topography. In summary, although the noise level of the SG at Jiufeng station in Wuhan has exhibited variation characteristics in some time periods of the last decade, no evidence that these phenomena are related to human activities has been found.

## Figures and Tables

**Table 1:** Data gaps in the gravity observations (UTC).

Data Gaps
2018-03-29T10:22:32 to 2018-04-04T04:41:58
2018-10-07T04:49:59 to 2018-10-11T12:17:00
2023-01-06T13:32:40 to 2023-01-16T08:46:00
2023-07-11T12:25:59 to 2023-07-12T00:00:00
2023-07-15T09:19:59 to 2023-07-16T00:00:00

## Data Availability

The data presented in this study are available from the corresponding author upon request. All of the data were processed and plotted using ObsPy and matplotlib.
